# Two new glaserite-type orthovanadates: Rb_2_KDy(VO_4_)_2_ and Cs_1.52_K_1.48_Gd(VO_4_)_2_

**DOI:** 10.1107/S2056989019008685

**Published:** 2019-06-21

**Authors:** Lotfi Rghioui, Lahcen El Ammari, Abderrazzak Assani, Mohamed Saadi

**Affiliations:** a Laboratoire de Spectroscopie, Modélisation Moléculaire, Matériaux, Nanomatériaux, Eau et Environnement (CERNE2D), Faculty of Sciences, Mohammed V University in Rabat, Avenue Ibn Batouta, BP 1014, Rabat, Morocco; b Laboratoire de Chimie Appliquée des Matériaux, Centre des Sciences des Matériaux, Faculty of Sciences, Mohammed V University in Rabat, Avenue Ibn Batouta, BP 1014, Rabat, Morocco

**Keywords:** crystal structure, crystal growth, X-ray diffraction, Rb_2_KDy(VO_4_)_2_, Cs_1.52_K_1.48_Gd(VO_4_)_2_, orthovanadates, IR and Raman spectroscopy

## Abstract

The crystal structures of dirubidium potassium dysprosium bis­(vanadate), Rb_2_KDy(VO_4_)_2_, and caesium potassium gadolinium bis­(vanadate), Cs_1.52_K_1.48_Gd(VO_4_)_2_, were solved from single-crystal X-ray diffraction data. Both compounds, synthesized by the reactive flux method, crystallize in the space group *P*  *m*1 with the glaserite structure type. VO_4_ tetra­hedra are linked to DyO_6_ or GdO_6_ octa­hedra by common vertices to form sheets stacking along the *c* axis. The large twelve-coordinate Cs^+^ or Rb^+^ cations are sandwiched between these layers in tunnels along the *a* and *b* axes, while the K^+^ cations, surrounded by ten oxygen atoms, are localized in cavities.

## Abstract 2

The title compounds have the glaserite structure type. The DyO_6_ or GdO_6_ octa­hedra share their three six vertices with six VO_4_ tetra­hedra, three of which are upward and the other three down. The remaining cations are localized between the sheets resulting from the tetra­hedra-octa­hedra linkage *via* common vertices.

## Chemical context   

Many studies have been devoted to phosphates, vanadates and arsenates with the general formula (*A*,*A*′)_3_ *Ln*(*X*O_4_)_2_ (*A*,*A*′ = alkaline elements, *Ln* = rare-earth element and *X* = P, V, As) because of their outstanding optical properties. This type of compound has numerous possible applications, such as their use in the production of low- and high-pressure mercury lamps or colour television screens (Hong & Chinn, 1976 ▸). It has been shown that these optical properties are enhanced by the presence of either a rare-earth element or an *X*O_4_ group and are determined by the fine details of the crystal structures of those materials (Benarafa *et al.*, 2005 ▸; Rghioui *et al.*, 1996 ▸, 1999 ▸, 2006 ▸). For instance, Parent *et al.* (1980 ▸) studied the luminescence phenomenon in Na_3_La_1–*x*_Nd_*x*_(PO_4_)_2_ and Na_3_La_1–*x*_Nd_*x*_(VO_4_)_2_ and measured the life time of the excited state 4*F* ^3/2^ as a function of the Nd^3+^ concentration. From a detailed examination of the emission and excitation spectra, Srivastava *et al.* (1990 ▸) highlighted an energy transfer from Ce^3+^ to the Tb^3+^ ion in the K_3_La_0.80_Ce_0.20_(PO_4_)_2_, K_3_La_0.80_Tb_0.20_(PO_4_)_2_ and K_3_La_1–*x*–*y*_Tb_x_Cey(PO_4_)_2_ phosphates. In addition, the band gaps and the life times of Ce^3+^ and Tb^3+^ were determined by Finke *et al.* (1992 ▸, 1994 ▸). The optical properties of the La atom in K_3_La(PO_4_)_2_; K_2_RbLa(PO_4_)_2_; Rb_2_KLa(PO_4_)_2_ and Rb_3_La(PO_4_)_2_ phosphates, investigated by FTIR and VUV spectroscopy, have allowed the determination of the values of band-gap energies for K_3_La(PO_4_)_2_ prepared by two different methods (Sasum *et al.*, 1997 ▸). In addition, Guzik *et al.* (2007 ▸) concluded that the emission phenomenon occurs from the charge transfer state in Na_3_Lu_1–*x*–*y*_Yb_*x*_(PO_4_)_2_ and Na_3_Y_1–*x*–*y*_Yb_*x*_(PO_4_)_2_ compounds. More recently, the optical properties of the Eu^3+^ ion were widely investigated in K_3_Eu(*X*O_4_)_2_ where *X* = P, As and V, K_2_YbHo_1–*x*–*y*_Eu_x_(PO_4_)_2_, K_2_Cs*Ln*(VO_4_)_2_ where *Ln* = La and Gd (Benarafa *et al.*, 2009 ▸; Rghioui *et al.*, 2015 ▸; Duke John David *et al.*, 2016 ▸; Tao *et al.*, 2014 ▸; Farmer *et al.*, 2014 ▸, 2016 ▸). In the case of K_3_Eu(*X*O_4_)_2_, a vibronic coupling mechanism was proposed to explain the process of europium emission observed under 647.1 nm excitation.

From a crystallographic point of view, the related (*A*,*A*′)_3_ *Ln*(XO_4_)_2_ compounds with *A*,*A*′ = K, Rb and Cs adopt three structure types. The first is a monoclinic system, space group *P*2_1_/*m*, represented by the phosphate K_3_Nd(PO_4_)_2_. The second one is trigonal, space group *P* 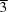, represented by K_3_Lu(PO_4_)_2_, while the third one is also trigonal but in space group *P* 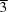 *m*1 and represented by the glaserite K_3_Na(SO_4_)_2_. The present work is a continuation of our structural investigations by X-ray diffraction of the (*A*,*A*′)_3_ *Ln*(*X*O_4_)_2_ system where *A*,*A*′ = K, Rb and Cs, *Ln* = rare earth and *X* = P, V, As (Rghioui *et al.*, 1999 ▸, 2002 ▸, 2007 ▸). The present paper reports the synthesis and the crystal structure determination of the title compounds by X-ray diffraction at room temperature and vibrational spectroscopy.

## Structural commentary   

**Figure 1:**
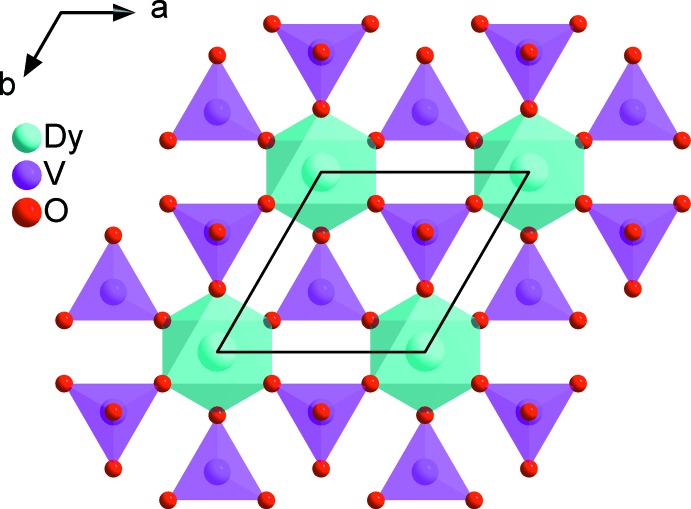
Layer of VO_4_ tetra­hedra linked to DyO_6_ octa­hedra by vertex sharing in the structure of Rb_2_KDy(VO_4_)_2_.

**Figure 2:**
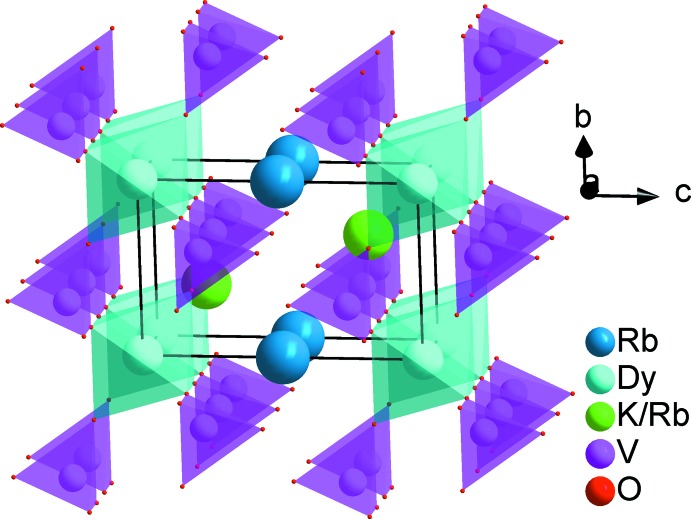
Three-dimensional view along the *a* axis of the crystal structure showing Rb^+^ (or Cs^+^) in the channels.

Dirubidium potassium dysprosium bis­(vanadate), Rb_2_KDy(VO_4_)_2_, and caesium potassium gadolinium bis­(vanadate), Cs_1.52_K_1.48_Gd(VO_4_)_2_, both compounds crystallize in the space group *P* 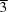 *m*1 with the common glaserite, K_3_Na(SO_4_)_2_, structure type (Moonre, 1973 ▸; Okada & Ossaka, 1980 ▸). The formulae determined by X-ray diffraction are consistent with the results of chemical analysis. In both structures, all atoms are in special positions of the *P* 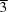 *m*1 space group, namely Dy1 in Wyckoff position 1*a* (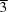 *m*), Rb1 in 1*b* (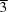 *m*), K1/Rb2, V1 and O2 in 2*d* (3*m*) and O1 in 6*i* (*m*). The structures of the two vanadates are built up from two independent VO_4_ tetra­hedra sharing an apex with DyO_6_ or GdO_6_ octa­hedra in such a way as to form a layer parallel to the ab plane, as shown in Fig. 1 ▸. Three of the six VO_4_ tetra­hedra surrounding each DyO_6_ or GdO_6_ octa­hedron are oriented upwards and the other three down. The concatenation of these polyhedra delimits large tunnels and cavities of site symmetry 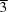 *m* and 3*m* in which are located rubidium and a statistical mixture of rubidium and potassium atoms (Fig. 2 ▸).

**Figure 3:**
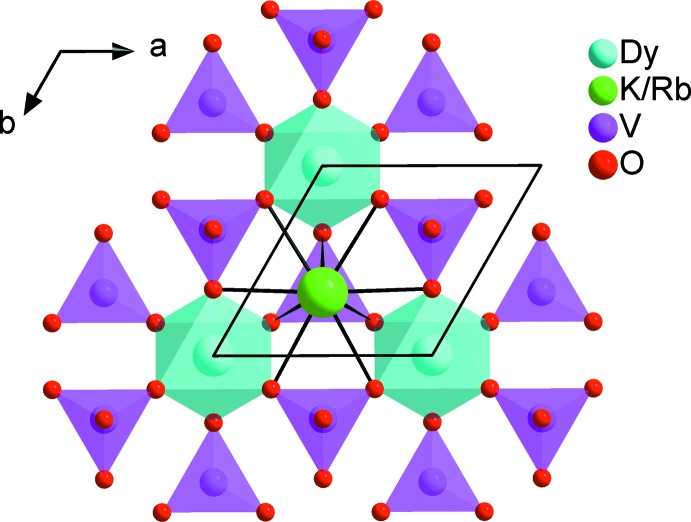
View along the *c* axis of a layer in the structure of the title compounds, showing the cavities in which the K/Rb^+^ (or K/Cs^+^) cations are located.

The coordination polyhedron of the mixed site is formed by ten oxygen atoms belonging to three edges, one face and one vertex of five VO_4_ tetra­hedra as shown in Fig. 3 ▸. The K/Rb—O distances range from 2.681 (8) to 3.312 (7) Å. The twelve oxygen atoms surrounding the rubidium atom form an irregular cubocta­hedron with Rb—O distances varying between 3.133 (2) and 3.4649 (3) Å. The main inter­atomic distances and angles are compatible with the values quoted in the literature (Gagné & Hawthorne, 2016 ▸; Gagné, 2018 ▸).

**Figure 4:**
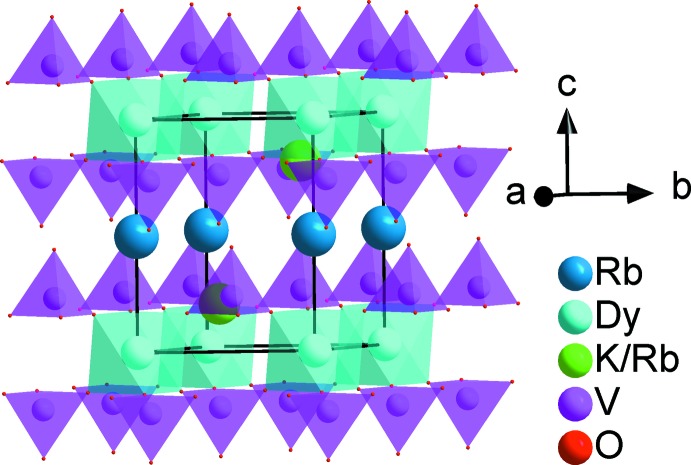
Three-dimensional view of the crystal structure showing Rb^+^ (or Cs^+^) cations between the layers stacked along the *c* axis.

**Figure 5:**
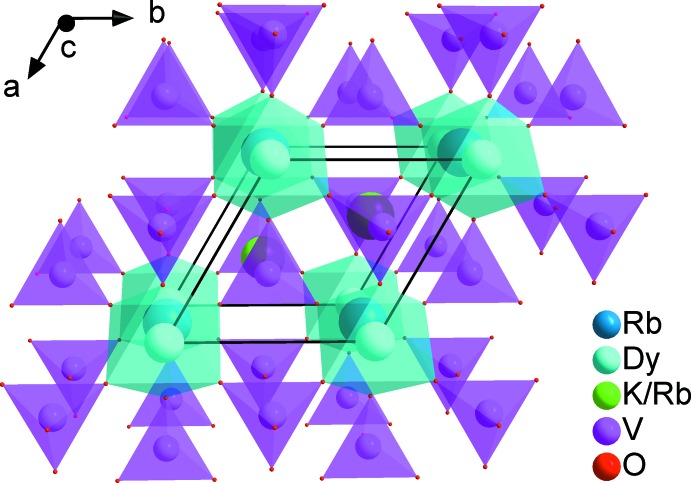
Three-dimensional perspective view along *c* axis of the crystal structure of Rb_2_KDy(VO_4_)_2_.

The three-dimensional structure consists of a basic tetra­hedral–octa­hedral framework, forming layers that stack along the *c*-axis direction, as shown in Fig. 4 ▸. In glaserite-like structures, the large cations are located between the layers in channels running along the *a*- and *b*-axis directions and the average size cations are located in the cavities (see Fig. 5 ▸).

## Vibrational spectroscopy   

**Figure 6:**
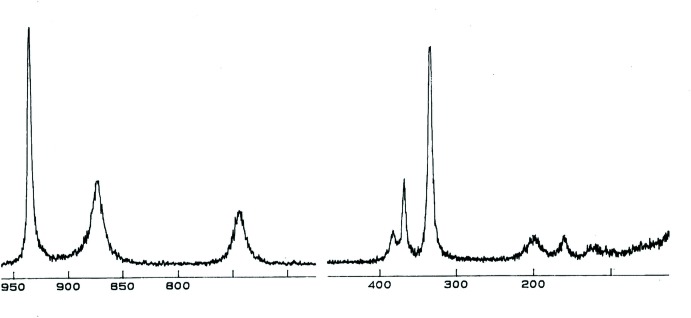
Raman spectrum of Rb_2_KDy(VO_4_)_2_.

The Raman and infrared spectra for Rb_2_KDy(VO_4_)_2_ are shown in Figs. 6 ▸ and 7 ▸, respectively. Their band assignments given in Table 1 ▸ are based on previous works for homologous vanadates (Rghioui *et al.*, 1999 ▸, 2012 ▸; Benarafa *et al.*, 2009 ▸). The stretching modes of (VO_4_)^3−^ anions are usually found in the region 950–700 cm^−1^. The peaks observed in the Raman spectrum at 935, 875 and 740 cm^−1^ as well as the corres­ponding bands in the infrared spectrum at 925, 830 and 755 cm^−1^ are all attributed to the symmetric (VO_4_)^3−^ and the asymmetric (VO_4_)^3−^ vibration. The bending vibrations of (VO_4_)^3−^ are seen in the range 390–310 cm^−1^. As in previous works (Rghioui *et al.*, 2012 ▸), the separation between the symmetric and asymmetric bending can not be identified in the vibrational spectra. The bands lying between 230 and 95 cm^−1^ in the spectra are assigned to the lattice vibrations. They are due to the VO_4_ rotation and to the VO_4_, K^+^, Rb^+^ and Dy^3+^ translation modes. A comparison of the Raman and infrared bands shows that they are not coincident. This fact confirms the centrosymmetric structure of Rb_2_KDy(VO_4_)_2_ vanadate.

## Synthesis and crystallization   

Single crystals of Rb_2_KDy(VO_4_)_2_ and Cs_1.52_K_1.48_Gd(VO_4_)_2_ were synthesized by the flux method using a mixture of K_2_CO_3_, Rb_2_CO_3_ (or Cs_2_CO_3_ for the Gd compound), Dy_2_O_3_ (or Gd_2_O_3_) and V_2_O_5_ corresponding to 1 mol of K_2_RbDy(VO_4_)_2_ (or Cs_1.52_K_1.48_Gd(VO_4_)_2_ and 1 mol of Rb_3_VO_4_ (or Cs_3_VO_4_). The reagents were ground in an agate mortar and placed in a platinum crucible. The temperature was raised slowly to 873 K and maintained for 24 h, permitting the carbonates to decompose. A second treatment at the melting temperature of 1273 K was performed, followed by slow cooling at a rate of 4 K h^−1^ to 673 K and then quickly to ambient temperature. Each thermal treatment was inter­spersed with grinding. The obtained product was then washed with distilled water in order to eliminate the flux. The resulting product contained single crystals of a suitable size for the X-ray diffraction study.

## Refinement   

Crystal data, data collection and structure refinement details are summarized in Table 2 ▸. In the refinement procedure, the substitutional occupation of the mixed sites was freely refined and restricted to the occupancy of one site for Cs_1.52_K_1.48_Gd(VO_4_)_2_ but restricted to 0.5:0.5 for Rb_2_KDy(VO_4_)_2_.

## Supplementary Material

Crystal structure: contains datablock(s) I, II, global. DOI: 10.1107/S2056989019008685/vn2149sup1.cif

Structure factors: contains datablock(s) I. DOI: 10.1107/S2056989019008685/vn2149Isup2.hkl

Structure factors: contains datablock(s) II. DOI: 10.1107/S2056989019008685/vn2149IIsup3.hkl

CCDC references: 1934723, 1934722

Additional supporting information:  crystallographic information; 3D view; checkCIF report

## Figures and Tables

**Figure 7:**
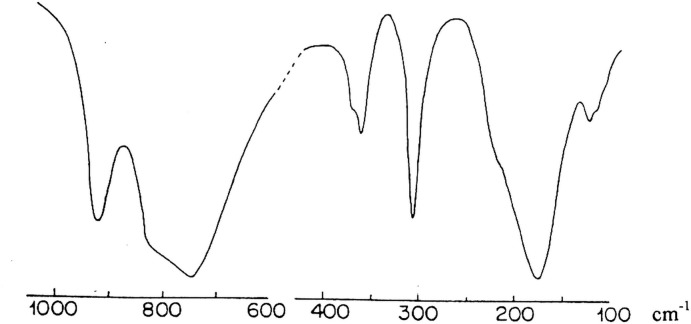
Infrared spectrum of Rb_2_KDy(VO_4_)_2_.

**Table 1:** Raman and Infrared band assignments (cm^−1^) for Rb_2_KDy(VO_4_)_2_

Raman	Infrared	Attribution
935	925	Stretching vibrations of VO_4_ groups
875	830	
740	755	
		
385	377	Deformation modes of VO_4_ groups
370	365	
340	311	
		
200	230	External modes
160	177	
125	130	
95	120	

**Table 2:** Experimental details

	Rb_2_KDy(VO_4_)_2_	Cs_1.52_K_1.48_Gd(VO_4_)_2_
Crystal data		
*M*_r_	602.42	646.74
Crystal system, space group	Trigonal, *P* 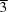 *m*1	Trigonal, *P* 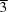 *m*1
Temperature (K)	296	296
*a*, *c* (Å)	5.9728 (1), 7.7780 (1)	6.0321 (1), 7.9821 (2)
*V* (Å^3^)	240.30 (1)	251.53 (1)
*Z*	1	1
Radiation type	Mo *K*α	Mo *K*α
μ (mm^−1^)	20.10	14.37
Crystal size (mm)	0.35 × 0.28 × 0.25	0.34 × 0.26 × 0.22

Data collection		
Diffractometer	Bruker X8 *APEX*	Bruker X8 *APEX*
Absorption correction	Multi-scan (*SADABS*; Krause *et al.*, 2015 ▸)	Multi-scan (*SADABS*; Krause *et al.*, 2015 ▸)
*T*_min_, *T*_max_	0.357, 0.749	0.639, 0.747
No. of measured, independent and observed [*I* > 2σ(*I*)] reflections	10549, 647, 631	14472, 678, 666
*R*_int_	0.049	0.038
(sin θ/λ)_max_ (Å^−1^)	0.926	0.925

Refinement		
*R*[*F* ^2^ > 2σ(*F* ^2^)], *wR*(*F* ^2^), *S*	0.016, 0.042, 1.11	0.010, 0.028, 1.09
No. of reflections	647	678
No. of parameters	24	25
Δρ_max_, Δρ_min_ (e Å^−3^)	1.35, −1.35	0.63, −0.94

Computer programs: *APEX2* and *SAINT-Plus* (Bruker, 2009 ▸), *SHELXT2014* (Sheldrick, 2015*a* ▸), *SHELXL2018* (Sheldrick, 2015*b* ▸), *ORTEP-3 for Windows* (Farrugia, 2012 ▸), *DIAMOND* (Brandenburg, 2006 ▸), *Mercury* (Macrae *et al.*, 2008 ▸) and *publCIF* (Westrip, 2010 ▸).

## supplementary crystallographic information

### Dirubidium potassium dysprosium bis(vanadate) (I) Crystal data

**Table:**

Rb_2_KDy(VO_4_)_2_	*D*_x_ = 4.163 Mg m^−^^3^
*M**_r_* = 602.42	Mo *K*α radiation, λ = 0.71073 Å
Trigonal, *P*3*m*1	Cell parameters from 647 reflections
*a* = 5.9728 (1) Å	θ = 2.6–41.1°
*c* = 7.7780 (1) Å	µ = 20.10 mm^−^^1^
*V* = 240.30 (1) Å^3^	*T* = 296 K
*Z* = 1	Block, colourless
*F*(000) = 269	0.35 × 0.28 × 0.25 mm

### Dirubidium potassium dysprosium bis(vanadate) (I) Data collection

**Table:**

Bruker X8 APEX diffractometer	647 independent reflections
Radiation source: fine-focus sealed tube	631 reflections with *I* > 2σ(*I*)
Graphite monochromator	*R*_int_ = 0.049
φ and ω scans	θ_max_ = 41.1°, θ_min_ = 2.6°
Absorption correction: multi-scan (SADABS; Krause *et al.*, 2015)	*h* = −11→11
*T*_min_ = 0.357, *T*_max_ = 0.749	*k* = −11→10
10549 measured reflections	*l* = −14→14

### Dirubidium potassium dysprosium bis(vanadate) (I) Refinement

**Table:**

Refinement on *F*^2^	0 restraints
Least-squares matrix: full	*w* = 1/[σ^2^(*F*_o_^2^) + (0.0239*P*)^2^ + 0.1252*P*] where *P* = (*F*_o_^2^ + 2*F*_c_^2^)/3
*R*[*F*^2^ > 2σ(*F*^2^)] = 0.016	(Δ/σ)_max_ < 0.001
*wR*(*F*^2^) = 0.042	Δρ_max_ = 1.35 e Å^−^^3^
*S* = 1.11	Δρ_min_ = −1.35 e Å^−^^3^
647 reflections	Extinction correction: SHELXL2018 (Sheldrick, 2015*b*), Fc^*^=kFc[1+0.001xFc^2^λ^3^/sin(2θ)]^-1/4^
24 parameters	Extinction coefficient: 0.0124 (15)

### Dirubidium potassium dysprosium bis(vanadate) (I) Special details

**Table:**

Geometry. All esds (except the esd in the dihedral angle between two l.s. planes) are estimated using the full covariance matrix. The cell esds are taken into account individually in the estimation of esds in distances, angles and torsion angles; correlations between esds in cell parameters are only used when they are defined by crystal symmetry. An approximate (isotropic) treatment of cell esds is used for estimating esds involving l.s. planes.

### Dirubidium potassium dysprosium bis(vanadate) (I) Fractional atomic coordinates and isotropic or equivalent isotropic displacement parameters (Å^2^)

**Table:**

	*x*	*y*	*z*	*U*_iso_*/*U*_eq_	Occ. (<1)
Rb1	0.000000	0.000000	0.500000	0.03556 (16)	
Dy1	0.000000	0.000000	0.000000	0.00791 (6)	
K1	0.333333	0.666667	0.8013 (9)	0.0098 (8)	0.5
Rb2	0.333333	0.666667	0.7969 (5)	0.0191 (6)	0.5
V1	0.333333	0.666667	0.24618 (6)	0.00765 (8)	
O1	0.17571 (17)	0.82429 (17)	0.1720 (3)	0.0273 (4)	
O2	0.333333	0.666667	0.4567 (4)	0.0389 (10)	

### Dirubidium potassium dysprosium bis(vanadate) (I) Atomic displacement parameters (Å^2^)

**Table:**

	*U*^11^	*U*^22^	*U*^33^	*U*^12^	*U*^13^	*U*^23^
Rb1	0.0463 (3)	0.0463 (3)	0.0141 (2)	0.02314 (13)	0.000	0.000
Dy1	0.00565 (6)	0.00565 (6)	0.01243 (8)	0.00282 (3)	0.000	0.000
K1	0.0082 (10)	0.0082 (10)	0.0128 (18)	0.0041 (5)	0.000	0.000
Rb2	0.0194 (7)	0.0194 (7)	0.0185 (12)	0.0097 (4)	0.000	0.000
V1	0.00766 (10)	0.00766 (10)	0.00763 (16)	0.00383 (5)	0.000	0.000
O1	0.0219 (5)	0.0219 (5)	0.0424 (10)	0.0141 (6)	−0.0081 (3)	0.0081 (3)
O2	0.0542 (15)	0.0542 (15)	0.0084 (10)	0.0271 (8)	0.000	0.000

### Dirubidium potassium dysprosium bis(vanadate) (I) Geometric parameters (Å, º)

**Table:**

Rb1—O1^i^	3.133 (2)	K1—O1^xvii^	2.9951 (5)
Rb1—O1^ii^	3.133 (2)	K1—O1^xviii^	2.9951 (6)
Rb1—O1^iii^	3.133 (2)	K1—O1^iii^	2.9951 (6)
Rb1—O1^iv^	3.133 (2)	K1—O1^xix^	2.9951 (5)
Rb1—O1^v^	3.133 (2)	K1—O1^i^	2.9951 (5)
Rb1—O1^vi^	3.133 (2)	K1—O1^xx^	3.312 (7)
Rb1—O2^vii^	3.4648 (3)	K1—O1^xxi^	3.312 (7)
Rb1—O2^viii^	3.4648 (3)	K1—O1^xxii^	3.312 (7)
Rb1—O2^ix^	3.4648 (3)	K1—V1^xx^	3.460 (7)
Rb1—O2	3.4648 (3)	K1—V1^vii^	3.4681 (8)
Rb1—O2^iii^	3.4649 (3)	Rb2—O2	2.647 (5)
Rb1—O2^iv^	3.4649 (3)	Rb2—O1^xvi^	2.9976 (4)
Dy1—O1^x^	2.2569 (17)	Rb2—O1^xvii^	2.9976 (4)
Dy1—O1^vi^	2.2569 (17)	Rb2—O1^xviii^	2.9976 (4)
Dy1—O1^xi^	2.2569 (17)	Rb2—O1^iii^	2.9976 (4)
Dy1—O1^iv^	2.2569 (17)	Rb2—O1^xix^	2.9976 (4)
Dy1—O1^xii^	2.2569 (17)	Rb2—O1^i^	2.9976 (4)
Dy1—O1^ii^	2.2569 (17)	Rb2—O1^xx^	3.342 (4)
Dy1—K1^xiii^	3.779 (3)	Rb2—O1^xxi^	3.342 (4)
Dy1—K1^vii^	3.779 (3)	Rb2—O1^xxii^	3.342 (4)
Dy1—K1^xiv^	3.779 (3)	Rb2—V1^vii^	3.4646 (4)
Dy1—K1^ix^	3.779 (3)	Rb2—V1^iii^	3.4647 (4)
Dy1—K1^xv^	3.779 (3)	V1—O2	1.637 (3)
Dy1—K1^iii^	3.779 (3)	V1—O1	1.7297 (16)
K1—O2	2.681 (8)	V1—O1^vi^	1.7297 (16)
K1—O1^xvi^	2.9951 (5)	V1—O1^xxiii^	1.7297 (16)
			
O1^i^—Rb1—O1^ii^	180.0	O1^xviii^—K1—V1^xx^	86.02 (15)
O1^i^—Rb1—O1^iii^	60.33 (5)	O1^iii^—K1—V1^xx^	86.02 (15)
O1^ii^—Rb1—O1^iii^	119.67 (5)	O1^xix^—K1—V1^xx^	86.02 (15)
O1^i^—Rb1—O1^iv^	119.67 (5)	O1^i^—K1—V1^xx^	86.02 (15)
O1^ii^—Rb1—O1^iv^	60.33 (5)	O1^xx^—K1—V1^xx^	29.49 (7)
O1^iii^—Rb1—O1^iv^	180.0	O1^xxi^—K1—V1^xx^	29.49 (7)
O1^i^—Rb1—O1^v^	60.33 (5)	O1^xxii^—K1—V1^xx^	29.49 (7)
O1^ii^—Rb1—O1^v^	119.67 (5)	O2—K1—V1^vii^	83.89 (12)
O1^iii^—Rb1—O1^v^	60.33 (5)	O1^xvi^—K1—V1^vii^	148.26 (4)
O1^iv^—Rb1—O1^v^	119.67 (5)	O1^xvii^—K1—V1^vii^	148.26 (4)
O1^i^—Rb1—O1^vi^	119.67 (5)	O1^xviii^—K1—V1^vii^	92.20 (4)
O1^ii^—Rb1—O1^vi^	60.33 (5)	O1^iii^—K1—V1^vii^	92.20 (4)
O1^iii^—Rb1—O1^vi^	119.67 (5)	O1^xix^—K1—V1^vii^	29.92 (3)
O1^iv^—Rb1—O1^vi^	60.33 (5)	O1^i^—K1—V1^vii^	29.92 (3)
O1^v^—Rb1—O1^vi^	180.0	O1^xx^—K1—V1^vii^	125.60 (19)
O1^i^—Rb1—O2^vii^	48.95 (6)	O1^xxi^—K1—V1^vii^	81.25 (8)
O1^ii^—Rb1—O2^vii^	131.05 (6)	O1^xxii^—K1—V1^vii^	81.25 (8)
O1^iii^—Rb1—O2^vii^	102.09 (5)	V1^xx^—K1—V1^vii^	96.11 (12)
O1^iv^—Rb1—O2^vii^	77.91 (5)	O2—Rb2—O1^xvi^	94.63 (9)
O1^v^—Rb1—O2^vii^	102.09 (5)	O2—Rb2—O1^xvii^	94.63 (9)
O1^vi^—Rb1—O2^vii^	77.91 (5)	O1^xvi^—Rb2—O1^xvii^	63.36 (7)
O1^i^—Rb1—O2^viii^	131.05 (6)	O2—Rb2—O1^xviii^	94.63 (9)
O1^ii^—Rb1—O2^viii^	48.95 (6)	O1^xvi^—Rb2—O1^xviii^	56.21 (7)
O1^iii^—Rb1—O2^viii^	77.91 (5)	O1^xvii^—Rb2—O1^xviii^	119.36 (3)
O1^iv^—Rb1—O2^viii^	102.09 (5)	O2—Rb2—O1^iii^	94.63 (9)
O1^v^—Rb1—O2^viii^	77.91 (5)	O1^xvi^—Rb2—O1^iii^	119.36 (3)
O1^vi^—Rb1—O2^viii^	102.09 (5)	O1^xvii^—Rb2—O1^iii^	56.21 (7)
O2^vii^—Rb1—O2^viii^	180.00 (11)	O1^xviii^—Rb2—O1^iii^	170.08 (18)
O1^i^—Rb1—O2^ix^	102.09 (5)	O2—Rb2—O1^xix^	94.63 (9)
O1^ii^—Rb1—O2^ix^	77.91 (5)	O1^xvi^—Rb2—O1^xix^	119.36 (3)
O1^iii^—Rb1—O2^ix^	102.09 (5)	O1^xvii^—Rb2—O1^xix^	170.08 (18)
O1^iv^—Rb1—O2^ix^	77.91 (5)	O1^xviii^—Rb2—O1^xix^	63.36 (7)
O1^v^—Rb1—O2^ix^	48.95 (6)	O1^iii^—Rb2—O1^xix^	119.36 (3)
O1^vi^—Rb1—O2^ix^	131.05 (6)	O2—Rb2—O1^i^	94.63 (9)
O2^vii^—Rb1—O2^ix^	119.065 (18)	O1^xvi^—Rb2—O1^i^	170.08 (18)
O2^viii^—Rb1—O2^ix^	60.935 (18)	O1^xvii^—Rb2—O1^i^	119.36 (3)
O1^i^—Rb1—O2	77.91 (5)	O1^xviii^—Rb2—O1^i^	119.36 (3)
O1^ii^—Rb1—O2	102.09 (5)	O1^iii^—Rb2—O1^i^	63.36 (7)
O1^iii^—Rb1—O2	77.91 (5)	O1^xix^—Rb2—O1^i^	56.21 (7)
O1^iv^—Rb1—O2	102.09 (5)	O2—Rb2—O1^xx^	150.80 (5)
O1^v^—Rb1—O2	131.05 (6)	O1^xvi^—Rb2—O1^xx^	61.07 (8)
O1^vi^—Rb1—O2	48.95 (6)	O1^xvii^—Rb2—O1^xx^	61.07 (8)
O2^vii^—Rb1—O2	60.935 (18)	O1^xviii^—Rb2—O1^xx^	85.09 (8)
O2^viii^—Rb1—O2	119.065 (18)	O1^iii^—Rb2—O1^xx^	85.09 (8)
O2^ix^—Rb1—O2	180.0	O1^xix^—Rb2—O1^xx^	110.99 (11)
O1^i^—Rb1—O2^iii^	102.09 (5)	O1^i^—Rb2—O1^xx^	110.99 (11)
O1^ii^—Rb1—O2^iii^	77.91 (5)	O2—Rb2—O1^xxi^	150.80 (5)
O1^iii^—Rb1—O2^iii^	48.95 (6)	O1^xvi^—Rb2—O1^xxi^	85.09 (8)
O1^iv^—Rb1—O2^iii^	131.05 (6)	O1^xvii^—Rb2—O1^xxi^	110.99 (11)
O1^v^—Rb1—O2^iii^	102.09 (5)	O1^xviii^—Rb2—O1^xxi^	61.07 (8)
O1^vi^—Rb1—O2^iii^	77.91 (5)	O1^iii^—Rb2—O1^xxi^	110.99 (11)
O2^vii^—Rb1—O2^iii^	119.064 (18)	O1^xix^—Rb2—O1^xxi^	61.07 (8)
O2^viii^—Rb1—O2^iii^	60.936 (18)	O1^i^—Rb2—O1^xxi^	85.09 (8)
O2^ix^—Rb1—O2^iii^	119.064 (18)	O1^xx^—Rb2—O1^xxi^	49.99 (8)
O2—Rb1—O2^iii^	60.935 (18)	O2—Rb2—O1^xxii^	150.80 (5)
O1^i^—Rb1—O2^iv^	77.91 (5)	O1^xvi^—Rb2—O1^xxii^	110.99 (11)
O1^ii^—Rb1—O2^iv^	102.09 (5)	O1^xvii^—Rb2—O1^xxii^	85.09 (8)
O1^iii^—Rb1—O2^iv^	131.05 (6)	O1^xviii^—Rb2—O1^xxii^	110.99 (11)
O1^iv^—Rb1—O2^iv^	48.95 (6)	O1^iii^—Rb2—O1^xxii^	61.07 (8)
O1^v^—Rb1—O2^iv^	77.91 (5)	O1^xix^—Rb2—O1^xxii^	85.09 (8)
O1^vi^—Rb1—O2^iv^	102.09 (5)	O1^i^—Rb2—O1^xxii^	61.07 (8)
O2^vii^—Rb1—O2^iv^	60.936 (18)	O1^xx^—Rb2—O1^xxii^	49.99 (8)
O2^viii^—Rb1—O2^iv^	119.064 (18)	O1^xxi^—Rb2—O1^xxii^	49.99 (8)
O2^ix^—Rb1—O2^iv^	60.936 (18)	O2—Rb2—V1^vii^	84.45 (7)
O2—Rb1—O2^iv^	119.065 (18)	O1^xvi^—Rb2—V1^vii^	148.32 (3)
O2^iii^—Rb1—O2^iv^	180.0	O1^xvii^—Rb2—V1^vii^	148.32 (3)
O1^x^—Dy1—O1^vi^	180.00 (12)	O1^xviii^—Rb2—V1^vii^	92.23 (3)
O1^x^—Dy1—O1^xi^	88.46 (9)	O1^iii^—Rb2—V1^vii^	92.23 (3)
O1^vi^—Dy1—O1^xi^	91.54 (9)	O1^xix^—Rb2—V1^vii^	29.95 (3)
O1^x^—Dy1—O1^iv^	91.54 (9)	O1^i^—Rb2—V1^vii^	29.95 (3)
O1^vi^—Dy1—O1^iv^	88.46 (9)	O1^xx^—Rb2—V1^vii^	124.76 (11)
O1^xi^—Dy1—O1^iv^	180.00 (9)	O1^xxi^—Rb2—V1^vii^	80.89 (5)
O1^x^—Dy1—O1^xii^	88.46 (9)	O1^xxii^—Rb2—V1^vii^	80.89 (5)
O1^vi^—Dy1—O1^xii^	91.54 (9)	O2—Rb2—V1^iii^	84.45 (7)
O1^xi^—Dy1—O1^xii^	88.46 (9)	O1^xvi^—Rb2—V1^iii^	92.23 (3)
O1^iv^—Dy1—O1^xii^	91.54 (9)	O1^xvii^—Rb2—V1^iii^	29.95 (3)
O1^x^—Dy1—O1^ii^	91.54 (9)	O1^xviii^—Rb2—V1^iii^	148.32 (3)
O1^vi^—Dy1—O1^ii^	88.46 (9)	O1^iii^—Rb2—V1^iii^	29.95 (3)
O1^xi^—Dy1—O1^ii^	91.54 (9)	O1^xix^—Rb2—V1^iii^	148.32 (3)
O1^iv^—Dy1—O1^ii^	88.46 (9)	O1^i^—Rb2—V1^iii^	92.23 (3)
O1^xii^—Dy1—O1^ii^	180.00 (10)	O1^xx^—Rb2—V1^iii^	80.89 (5)
O1^x^—Dy1—K1^xiii^	52.42 (5)	O1^xxi^—Rb2—V1^iii^	124.76 (11)
O1^vi^—Dy1—K1^xiii^	127.58 (5)	O1^xxii^—Rb2—V1^iii^	80.89 (5)
O1^xi^—Dy1—K1^xiii^	52.42 (5)	V1^vii^—Rb2—V1^iii^	119.07 (2)
O1^iv^—Dy1—K1^xiii^	127.58 (5)	O2—V1—O1	109.49 (8)
O1^xii^—Dy1—K1^xiii^	119.51 (12)	O2—V1—O1^vi^	109.49 (8)
O1^ii^—Dy1—K1^xiii^	60.49 (12)	O1—V1—O1^vi^	109.45 (8)
O1^x^—Dy1—K1^vii^	127.58 (5)	O2—V1—O1^xxiii^	109.49 (8)
O1^vi^—Dy1—K1^vii^	52.42 (5)	O1—V1—O1^xxiii^	109.45 (8)
O1^xi^—Dy1—K1^vii^	127.58 (5)	O1^vi^—V1—O1^xxiii^	109.45 (8)
O1^iv^—Dy1—K1^vii^	52.42 (5)	O2—V1—K1^xiv^	180.0
O1^xii^—Dy1—K1^vii^	60.49 (12)	O1—V1—K1^xiv^	70.51 (8)
O1^ii^—Dy1—K1^vii^	119.51 (12)	O1^vi^—V1—K1^xiv^	70.51 (8)
K1^xiii^—Dy1—K1^vii^	180.0	O1^xxiii^—V1—K1^xiv^	70.51 (8)
O1^x^—Dy1—K1^xiv^	119.51 (12)	O2—V1—K1^vii^	96.11 (12)
O1^vi^—Dy1—K1^xiv^	60.49 (12)	O1—V1—K1^vii^	154.40 (15)
O1^xi^—Dy1—K1^xiv^	52.42 (5)	O1^vi^—V1—K1^vii^	59.72 (4)
O1^iv^—Dy1—K1^xiv^	127.58 (5)	O1^xxiii^—V1—K1^vii^	59.72 (4)
O1^xii^—Dy1—K1^xiv^	52.42 (5)	K1^xiv^—V1—K1^vii^	83.89 (12)
O1^ii^—Dy1—K1^xiv^	127.58 (5)	O2—V1—K1^iii^	96.11 (12)
K1^xiii^—Dy1—K1^xiv^	104.42 (12)	O1—V1—K1^iii^	59.72 (4)
K1^vii^—Dy1—K1^xiv^	75.58 (12)	O1^vi^—V1—K1^iii^	59.72 (4)
O1^x^—Dy1—K1^ix^	60.49 (12)	O1^xxiii^—V1—K1^iii^	154.40 (15)
O1^vi^—Dy1—K1^ix^	119.51 (12)	K1^xiv^—V1—K1^iii^	83.89 (12)
O1^xi^—Dy1—K1^ix^	127.58 (5)	K1^vii^—V1—K1^iii^	118.88 (4)
O1^iv^—Dy1—K1^ix^	52.42 (5)	O2—V1—K1^xviii^	96.11 (12)
O1^xii^—Dy1—K1^ix^	127.58 (5)	O1—V1—K1^xviii^	59.72 (4)
O1^ii^—Dy1—K1^ix^	52.42 (5)	O1^vi^—V1—K1^xviii^	154.40 (15)
K1^xiii^—Dy1—K1^ix^	75.58 (12)	O1^xxiii^—V1—K1^xviii^	59.72 (4)
K1^vii^—Dy1—K1^ix^	104.42 (12)	K1^xiv^—V1—K1^xviii^	83.89 (12)
K1^xiv^—Dy1—K1^ix^	180.0	K1^vii^—V1—K1^xviii^	118.88 (5)
O1^x^—Dy1—K1^xv^	52.42 (5)	K1^iii^—V1—K1^xviii^	118.88 (4)
O1^vi^—Dy1—K1^xv^	127.58 (5)	O2—V1—Rb1^xxiv^	60.209 (6)
O1^xi^—Dy1—K1^xv^	119.51 (12)	O1—V1—Rb1^xxiv^	49.28 (8)
O1^iv^—Dy1—K1^xv^	60.49 (12)	O1^vi^—V1—Rb1^xxiv^	125.09 (3)
O1^xii^—Dy1—K1^xv^	52.42 (5)	O1^xxiii^—V1—Rb1^xxiv^	125.09 (3)
O1^ii^—Dy1—K1^xv^	127.58 (5)	K1^xiv^—V1—Rb1^xxiv^	119.792 (6)
K1^xiii^—Dy1—K1^xv^	104.42 (12)	K1^vii^—V1—Rb1^xxiv^	156.32 (12)
K1^vii^—Dy1—K1^xv^	75.58 (12)	K1^iii^—V1—Rb1^xxiv^	67.75 (7)
K1^xiv^—Dy1—K1^xv^	104.42 (12)	K1^xviii^—V1—Rb1^xxiv^	67.75 (7)
K1^ix^—Dy1—K1^xv^	75.58 (12)	O2—V1—Rb1^xxv^	60.209 (6)
O1^x^—Dy1—K1^iii^	127.58 (5)	O1—V1—Rb1^xxv^	125.09 (3)
O1^vi^—Dy1—K1^iii^	52.42 (5)	O1^vi^—V1—Rb1^xxv^	125.09 (3)
O1^xi^—Dy1—K1^iii^	60.49 (12)	O1^xxiii^—V1—Rb1^xxv^	49.28 (8)
O1^iv^—Dy1—K1^iii^	119.51 (12)	K1^xiv^—V1—Rb1^xxv^	119.791 (6)
O1^xii^—Dy1—K1^iii^	127.58 (5)	K1^vii^—V1—Rb1^xxv^	67.76 (7)
O1^ii^—Dy1—K1^iii^	52.42 (5)	K1^iii^—V1—Rb1^xxv^	156.32 (12)
K1^xiii^—Dy1—K1^iii^	75.58 (12)	K1^xviii^—V1—Rb1^xxv^	67.76 (7)
K1^vii^—Dy1—K1^iii^	104.42 (12)	Rb1^xxiv^—V1—Rb1^xxv^	97.454 (8)
K1^xiv^—Dy1—K1^iii^	75.58 (12)	O2—V1—Rb1	60.209 (6)
K1^ix^—Dy1—K1^iii^	104.42 (12)	O1—V1—Rb1	125.09 (3)
K1^xv^—Dy1—K1^iii^	180.0	O1^vi^—V1—Rb1	49.28 (8)
O2—K1—O1^xvi^	93.98 (15)	O1^xxiii^—V1—Rb1	125.09 (3)
O2—K1—O1^xvii^	93.98 (15)	K1^xiv^—V1—Rb1	119.791 (6)
O1^xvi^—K1—O1^xvii^	63.42 (7)	K1^vii^—V1—Rb1	67.76 (7)
O2—K1—O1^xviii^	93.98 (15)	K1^iii^—V1—Rb1	67.76 (7)
O1^xvi^—K1—O1^xviii^	56.26 (7)	K1^xviii^—V1—Rb1	156.32 (12)
O1^xvii^—K1—O1^xviii^	119.52 (4)	Rb1^xxiv^—V1—Rb1	97.454 (8)
O2—K1—O1^iii^	93.98 (15)	Rb1^xxv^—V1—Rb1	97.454 (8)
O1^xvi^—K1—O1^iii^	119.52 (4)	O2—V1—Rb2	0.000 (1)
O1^xvii^—K1—O1^iii^	56.26 (7)	O1—V1—Rb2	109.49 (8)
O1^xviii^—K1—O1^iii^	171.3 (3)	O1^vi^—V1—Rb2	109.49 (8)
O2—K1—O1^xix^	93.98 (15)	O1^xxiii^—V1—Rb2	109.49 (8)
O1^xvi^—K1—O1^xix^	119.52 (4)	K1^xiv^—V1—Rb2	180.0
O1^xvii^—K1—O1^xix^	171.3 (3)	K1^vii^—V1—Rb2	96.11 (12)
O1^xviii^—K1—O1^xix^	63.42 (7)	K1^iii^—V1—Rb2	96.11 (12)
O1^iii^—K1—O1^xix^	119.52 (4)	K1^xviii^—V1—Rb2	96.11 (12)
O2—K1—O1^i^	93.98 (15)	Rb1^xxiv^—V1—Rb2	60.209 (6)
O1^xvi^—K1—O1^i^	171.3 (3)	Rb1^xxv^—V1—Rb2	60.209 (6)
O1^xvii^—K1—O1^i^	119.52 (4)	Rb1—V1—Rb2	60.209 (6)
O1^xviii^—K1—O1^i^	119.52 (4)	V1—O1—Dy1^xxiv^	163.14 (14)
O1^iii^—K1—O1^i^	63.42 (7)	V1—O1—K1^xviii^	90.36 (6)
O1^xix^—K1—O1^i^	56.26 (7)	Dy1^xxiv^—O1—K1^xviii^	90.91 (9)
O2—K1—O1^xx^	150.51 (7)	V1—O1—K1^iii^	90.36 (6)
O1^xvi^—K1—O1^xx^	61.46 (10)	Dy1^xxiv^—O1—K1^iii^	90.91 (9)
O1^xvii^—K1—O1^xx^	61.46 (10)	K1^xviii^—O1—K1^iii^	171.3 (3)
O1^xviii^—K1—O1^xx^	85.65 (13)	V1—O1—Rb1^xxiv^	105.98 (10)
O1^iii^—K1—O1^xx^	85.65 (13)	Dy1^xxiv^—O1—Rb1^xxiv^	90.88 (6)
O1^xix^—K1—O1^xx^	111.86 (19)	K1^xviii^—O1—Rb1^xxiv^	85.72 (12)
O1^i^—K1—O1^xx^	111.86 (19)	K1^iii^—O1—Rb1^xxiv^	85.72 (12)
O2—K1—O1^xxi^	150.51 (7)	V1—O1—K1^xiv^	80.00 (10)
O1^xvi^—K1—O1^xxi^	85.65 (13)	Dy1^xxiv^—O1—K1^xiv^	83.14 (9)
O1^xvii^—K1—O1^xxi^	111.86 (19)	K1^xviii^—O1—K1^xiv^	94.35 (13)
O1^xviii^—K1—O1^xxi^	61.46 (10)	K1^iii^—O1—K1^xiv^	94.35 (13)
O1^iii^—K1—O1^xxi^	111.86 (19)	Rb1^xxiv^—O1—K1^xiv^	174.02 (9)
O1^xix^—K1—O1^xxi^	61.46 (10)	V1—O2—Rb2	180.0
O1^i^—K1—O1^xxi^	85.65 (13)	V1—O2—K1	180.0
O1^xx^—K1—O1^xxi^	50.47 (12)	Rb2—O2—K1	0.000 (1)
O2—K1—O1^xxii^	150.51 (7)	V1—O2—Rb1^xxiv^	95.58 (5)
O1^xvi^—K1—O1^xxii^	111.86 (19)	Rb2—O2—Rb1^xxiv^	84.42 (5)
O1^xvii^—K1—O1^xxii^	85.65 (13)	K1—O2—Rb1^xxiv^	84.42 (5)
O1^xviii^—K1—O1^xxii^	111.86 (19)	V1—O2—Rb1^xxv^	95.58 (5)
O1^iii^—K1—O1^xxii^	61.46 (10)	Rb2—O2—Rb1^xxv^	84.42 (5)
O1^xix^—K1—O1^xxii^	85.65 (13)	K1—O2—Rb1^xxv^	84.42 (5)
O1^i^—K1—O1^xxii^	61.46 (10)	Rb1^xxiv^—O2—Rb1^xxv^	119.065 (18)
O1^xx^—K1—O1^xxii^	50.47 (12)	V1—O2—Rb1	95.58 (5)
O1^xxi^—K1—O1^xxii^	50.47 (12)	Rb2—O2—Rb1	84.42 (5)
O2—K1—V1^xx^	180.0	K1—O2—Rb1	84.42 (5)
O1^xvi^—K1—V1^xx^	86.02 (15)	Rb1^xxiv^—O2—Rb1	119.065 (18)
O1^xvii^—K1—V1^xx^	86.02 (15)	Rb1^xxv^—O2—Rb1	119.065 (18)

Symmetry codes: (i) *x*−*y*+1, *x*, −*z*+1; (ii) −*x*+*y*−1, −*x*, *z*; (iii) −*x*, −*y*+1, −*z*+1; (iv) *x*, *y*−1, *z*; (v) *y*−1, −*x*+*y*−1, −*z*+1; (vi) −*y*+1, *x*−*y*+1, *z*; (vii) −*x*+1, −*y*+1, −*z*+1; (viii) *x*−1, *y*−1, *z*; (ix) −*x*, −*y*, −*z*+1; (x) *y*−1, −*x*+*y*−1, −*z*; (xi) −*x*, −*y*+1, −*z*; (xii) *x*−*y*+1, *x*, −*z*; (xiii) *x*−1, *y*−1, *z*−1; (xiv) *x*, *y*, *z*−1; (xv) *x*, *y*−1, *z*−1; (xvi) *x*−*y*+1, *x*+1, −*z*+1; (xvii) *y*−1, −*x*+*y*, −*z*+1; (xviii) −*x*+1, −*y*+2, −*z*+1; (xix) *y*, −*x*+*y*, −*z*+1; (xx) *x*, *y*, *z*+1; (xxi) −*x*+*y*, −*x*+1, *z*+1; (xxii) −*y*+1, *x*−*y*+1, *z*+1; (xxiii) −*x*+*y*, −*x*+1, *z*; (xxiv) *x*, *y*+1, *z*; (xxv) *x*+1, *y*+1, *z*.

### Caesium potassium gadolinium bis(vanadate) (II) Crystal data

**Table:**

Cs_1.52_K_1.48_Gd(VO_4_)_2_	*D*_x_ = 4.270 Mg m^−^^3^
*M**_r_* = 646.74	Mo *K*α radiation, λ = 0.71073 Å
Trigonal, *P*3*m*1	Cell parameters from 678 reflections
*a* = 6.0321 (1) Å	θ = 3.9–41.1°
*c* = 7.9821 (2) Å	µ = 14.37 mm^−^^1^
*V* = 251.53 (1) Å^3^	*T* = 296 K
*Z* = 1	Block, colourless
*F*(000) = 286	0.34 × 0.26 × 0.22 mm

### Caesium potassium gadolinium bis(vanadate) (II) Data collection

**Table:**

Bruker X8 APEX diffractometer	678 independent reflections
Radiation source: fine-focus sealed tube	666 reflections with *I* > 2σ(*I*)
Graphite monochromator	*R*_int_ = 0.038
φ and ω scans	θ_max_ = 41.1°, θ_min_ = 3.9°
Absorption correction: multi-scan (SADABS; Krause *et al.*, 2015)	*h* = −11→11
*T*_min_ = 0.639, *T*_max_ = 0.747	*k* = −9→11
14472 measured reflections	*l* = −14→14

### Caesium potassium gadolinium bis(vanadate) (II) Refinement

**Table:**

Refinement on *F*^2^	0 restraints
Least-squares matrix: full	*w* = 1/[σ^2^(*F*_o_^2^) + (0.014*P*)^2^ + 0.0871*P*] where *P* = (*F*_o_^2^ + 2*F*_c_^2^)/3
*R*[*F*^2^ > 2σ(*F*^2^)] = 0.010	(Δ/σ)_max_ < 0.001
*wR*(*F*^2^) = 0.028	Δρ_max_ = 0.63 e Å^−^^3^
*S* = 1.09	Δρ_min_ = −0.94 e Å^−^^3^
678 reflections	Extinction correction: SHELXL2018 (Sheldrick, 2015*b*), Fc^*^=kFc[1+0.001xFc^2^λ^3^/sin(2θ)]^-1/4^
25 parameters	Extinction coefficient: 0.0022 (6)

### Caesium potassium gadolinium bis(vanadate) (II) Special details

**Table:**

Geometry. All esds (except the esd in the dihedral angle between two l.s. planes) are estimated using the full covariance matrix. The cell esds are taken into account individually in the estimation of esds in distances, angles and torsion angles; correlations between esds in cell parameters are only used when they are defined by crystal symmetry. An approximate (isotropic) treatment of cell esds is used for estimating esds involving l.s. planes.

### Caesium potassium gadolinium bis(vanadate) (II) Fractional atomic coordinates and isotropic or equivalent isotropic displacement parameters (Å^2^)

**Table:**

	*x*	*y*	*z*	*U*_iso_*/*U*_eq_	Occ. (<1)
Cs1	0.000000	0.000000	0.500000	0.02453 (5)	
Gd1	0.000000	0.000000	0.000000	0.00899 (4)	
K1	0.333333	0.666667	0.7877 (3)	0.0113 (5)	0.7404 (18)
Cs2	0.333333	0.666667	0.7867 (4)	0.0230 (8)	0.2597 (18)
V1	0.333333	0.666667	0.23977 (4)	0.00899 (5)	
O1	0.17704 (10)	0.82296 (10)	0.16910 (16)	0.0265 (2)	
O2	0.333333	0.666667	0.4470 (3)	0.0317 (5)	

### Caesium potassium gadolinium bis(vanadate) (II) Atomic displacement parameters (Å^2^)

**Table:**

	*U*^11^	*U*^22^	*U*^33^	*U*^12^	*U*^13^	*U*^23^
Cs1	0.02979 (7)	0.02979 (7)	0.01402 (8)	0.01489 (3)	0.000	0.000
Gd1	0.00627 (4)	0.00627 (4)	0.01444 (6)	0.00313 (2)	0.000	0.000
K1	0.0091 (6)	0.0091 (6)	0.0156 (9)	0.0046 (3)	0.000	0.000
Cs2	0.0200 (9)	0.0200 (9)	0.0288 (14)	0.0100 (5)	0.000	0.000
V1	0.00756 (7)	0.00756 (7)	0.01186 (11)	0.00378 (3)	0.000	0.000
O1	0.0244 (4)	0.0244 (4)	0.0379 (6)	0.0177 (4)	−0.00613 (19)	0.00613 (19)
O2	0.0404 (7)	0.0404 (7)	0.0144 (7)	0.0202 (4)	0.000	0.000

### Caesium potassium gadolinium bis(vanadate) (II) Geometric parameters (Å, º)

**Table:**

Cs1—O1^i^	3.2245 (13)	K1—O1^xvii^	3.0377 (4)
Cs1—O1^ii^	3.2245 (13)	K1—O1^xviii^	3.0377 (4)
Cs1—O1^iii^	3.2245 (13)	K1—O1^iii^	3.0377 (4)
Cs1—O1^iv^	3.2245 (13)	K1—O1^xix^	3.0377 (4)
Cs1—O1^v^	3.2245 (13)	K1—O1^i^	3.0377 (4)
Cs1—O1^vi^	3.2245 (13)	K1—V1^vii^	3.4895 (2)
Cs1—O2^vii^	3.5082 (3)	K1—V1^iii^	3.4895 (2)
Cs1—O2^viii^	3.5082 (3)	K1—V1^xviii^	3.4895 (2)
Cs1—O2^ix^	3.5082 (3)	K1—V1^xx^	3.609 (3)
Cs1—O2	3.5083 (3)	Cs2—O2	2.712 (4)
Cs1—O2^iii^	3.5083 (3)	Cs2—O1^xvi^	3.0386 (4)
Cs1—O2^iv^	3.5083 (2)	Cs2—O1^xvii^	3.0386 (4)
Gd1—O1^x^	2.2898 (10)	Cs2—O1^xviii^	3.0386 (4)
Gd1—O1^vi^	2.2898 (10)	Cs2—O1^iii^	3.0386 (4)
Gd1—O1^xi^	2.2898 (10)	Cs2—O1^xix^	3.0386 (4)
Gd1—O1^iv^	2.2898 (10)	Cs2—O1^i^	3.0386 (4)
Gd1—O1^xii^	2.2898 (10)	Cs2—O1^xx^	3.462 (3)
Gd1—O1^ii^	2.2898 (10)	Cs2—O1^xxi^	3.462 (3)
Gd1—K1^xiii^	3.8732 (12)	Cs2—O1^xxii^	3.462 (3)
Gd1—K1^vii^	3.8732 (12)	Cs2—V1^vii^	3.4890 (2)
Gd1—K1^xiv^	3.8731 (12)	Cs2—V1^iii^	3.4890 (2)
Gd1—K1^ix^	3.8731 (12)	V1—O2	1.654 (2)
Gd1—K1^xv^	3.8732 (12)	V1—O1	1.7276 (10)
Gd1—K1^iii^	3.8732 (12)	V1—O1^vi^	1.7276 (10)
K1—O2	2.719 (3)	V1—O1^xxiii^	1.7276 (10)
K1—O1^xvi^	3.0377 (4)		
			
O1^i^—Cs1—O1^ii^	180.0	O1^xvi^—K1—V1^xx^	83.48 (6)
O1^i^—Cs1—O1^iii^	59.57 (3)	O1^xvii^—K1—V1^xx^	83.48 (6)
O1^ii^—Cs1—O1^iii^	120.43 (3)	O1^xviii^—K1—V1^xx^	83.48 (6)
O1^i^—Cs1—O1^iv^	120.43 (3)	O1^iii^—K1—V1^xx^	83.48 (6)
O1^ii^—Cs1—O1^iv^	59.57 (3)	O1^xix^—K1—V1^xx^	83.48 (6)
O1^iii^—Cs1—O1^iv^	180.0	O1^i^—K1—V1^xx^	83.48 (6)
O1^i^—Cs1—O1^v^	59.57 (3)	V1^vii^—K1—V1^xx^	93.60 (4)
O1^ii^—Cs1—O1^v^	120.43 (3)	V1^iii^—K1—V1^xx^	93.60 (4)
O1^iii^—Cs1—O1^v^	59.57 (3)	V1^xviii^—K1—V1^xx^	93.60 (4)
O1^iv^—Cs1—O1^v^	120.43 (3)	O2—K1—Gd1^xxiv^	115.95 (4)
O1^i^—Cs1—O1^vi^	120.43 (3)	O1^xvi^—K1—Gd1^xxiv^	36.21 (2)
O1^ii^—Cs1—O1^vi^	59.57 (3)	O1^xvii^—K1—Gd1^xxiv^	36.21 (2)
O1^iii^—Cs1—O1^vi^	120.43 (3)	O1^xviii^—K1—Gd1^xxiv^	85.31 (3)
O1^iv^—Cs1—O1^vi^	59.57 (3)	O1^iii^—K1—Gd1^xxiv^	85.31 (3)
O1^v^—Cs1—O1^vi^	180.0	O1^xix^—K1—Gd1^xxiv^	137.69 (7)
O1^i^—Cs1—O2^vii^	48.07 (4)	O1^i^—K1—Gd1^xxiv^	137.69 (7)
O1^ii^—Cs1—O2^vii^	131.93 (4)	V1^vii^—K1—Gd1^xxiv^	157.65 (8)
O1^iii^—Cs1—O2^vii^	100.71 (3)	V1^iii^—K1—Gd1^xxiv^	65.087 (12)
O1^iv^—Cs1—O2^vii^	79.29 (3)	V1^xviii^—K1—Gd1^xxiv^	65.087 (12)
O1^v^—Cs1—O2^vii^	100.71 (3)	V1^xx^—K1—Gd1^xxiv^	64.05 (4)
O1^vi^—Cs1—O2^vii^	79.29 (3)	O2—Cs2—O1^xvi^	96.67 (7)
O1^i^—Cs1—O2^viii^	131.93 (4)	O2—Cs2—O1^xvii^	96.67 (7)
O1^ii^—Cs1—O2^viii^	48.07 (4)	O1^xvi^—Cs2—O1^xvii^	63.63 (4)
O1^iii^—Cs1—O2^viii^	79.29 (3)	O2—Cs2—O1^xviii^	96.67 (7)
O1^iv^—Cs1—O2^viii^	100.71 (3)	O1^xvi^—Cs2—O1^xviii^	55.47 (4)
O1^v^—Cs1—O2^viii^	79.29 (3)	O1^xvii^—Cs2—O1^xviii^	118.67 (3)
O1^vi^—Cs1—O2^viii^	100.71 (3)	O2—Cs2—O1^iii^	96.67 (7)
O2^vii^—Cs1—O2^viii^	180.0	O1^xvi^—Cs2—O1^iii^	118.67 (3)
O1^i^—Cs1—O2^ix^	100.71 (3)	O1^xvii^—Cs2—O1^iii^	55.47 (4)
O1^ii^—Cs1—O2^ix^	79.29 (3)	O1^xviii^—Cs2—O1^iii^	166.04 (13)
O1^iii^—Cs1—O2^ix^	100.71 (3)	O2—Cs2—O1^xix^	96.67 (7)
O1^iv^—Cs1—O2^ix^	79.29 (3)	O1^xvi^—Cs2—O1^xix^	118.67 (3)
O1^v^—Cs1—O2^ix^	48.07 (4)	O1^xvii^—Cs2—O1^xix^	166.04 (13)
O1^vi^—Cs1—O2^ix^	131.93 (4)	O1^xviii^—Cs2—O1^xix^	63.63 (4)
O2^vii^—Cs1—O2^ix^	118.567 (13)	O1^iii^—Cs2—O1^xix^	118.67 (3)
O2^viii^—Cs1—O2^ix^	61.433 (14)	O2—Cs2—O1^i^	96.67 (7)
O1^i^—Cs1—O2	79.29 (3)	O1^xvi^—Cs2—O1^i^	166.04 (13)
O1^ii^—Cs1—O2	100.71 (3)	O1^xvii^—Cs2—O1^i^	118.67 (3)
O1^iii^—Cs1—O2	79.29 (3)	O1^xviii^—Cs2—O1^i^	118.67 (3)
O1^iv^—Cs1—O2	100.71 (3)	O1^iii^—Cs2—O1^i^	63.63 (4)
O1^v^—Cs1—O2	131.93 (4)	O1^xix^—Cs2—O1^i^	55.47 (4)
O1^vi^—Cs1—O2	48.07 (4)	O2—Cs2—O1^xx^	151.86 (3)
O2^vii^—Cs1—O2	61.434 (13)	O1^xvi^—Cs2—O1^xx^	60.03 (5)
O2^viii^—Cs1—O2	118.566 (13)	O1^xvii^—Cs2—O1^xx^	60.03 (5)
O2^ix^—Cs1—O2	180.0	O1^xviii^—Cs2—O1^xx^	83.15 (6)
O1^i^—Cs1—O2^iii^	100.71 (3)	O1^iii^—Cs2—O1^xx^	83.15 (6)
O1^ii^—Cs1—O2^iii^	79.29 (3)	O1^xix^—Cs2—O1^xx^	108.15 (8)
O1^iii^—Cs1—O2^iii^	48.07 (4)	O1^i^—Cs2—O1^xx^	108.15 (8)
O1^iv^—Cs1—O2^iii^	131.93 (4)	O2—Cs2—O1^xxi^	151.86 (3)
O1^v^—Cs1—O2^iii^	100.71 (3)	O1^xvi^—Cs2—O1^xxi^	83.15 (6)
O1^vi^—Cs1—O2^iii^	79.29 (3)	O1^xvii^—Cs2—O1^xxi^	108.15 (8)
O2^vii^—Cs1—O2^iii^	118.565 (14)	O1^xviii^—Cs2—O1^xxi^	60.03 (5)
O2^viii^—Cs1—O2^iii^	61.435 (13)	O1^iii^—Cs2—O1^xxi^	108.15 (8)
O2^ix^—Cs1—O2^iii^	118.565 (14)	O1^xix^—Cs2—O1^xxi^	60.03 (5)
O2—Cs1—O2^iii^	61.434 (13)	O1^i^—Cs2—O1^xxi^	83.15 (6)
O1^i^—Cs1—O2^iv^	79.29 (3)	O1^xx^—Cs2—O1^xxi^	48.22 (5)
O1^ii^—Cs1—O2^iv^	100.71 (3)	O2—Cs2—O1^xxii^	151.86 (3)
O1^iii^—Cs1—O2^iv^	131.93 (4)	O1^xvi^—Cs2—O1^xxii^	108.15 (8)
O1^iv^—Cs1—O2^iv^	48.07 (4)	O1^xvii^—Cs2—O1^xxii^	83.15 (6)
O1^v^—Cs1—O2^iv^	79.29 (3)	O1^xviii^—Cs2—O1^xxii^	108.15 (8)
O1^vi^—Cs1—O2^iv^	100.71 (3)	O1^iii^—Cs2—O1^xxii^	60.03 (5)
O2^vii^—Cs1—O2^iv^	61.435 (13)	O1^xix^—Cs2—O1^xxii^	83.15 (6)
O2^viii^—Cs1—O2^iv^	118.565 (14)	O1^i^—Cs2—O1^xxii^	60.03 (5)
O2^ix^—Cs1—O2^iv^	61.435 (13)	O1^xx^—Cs2—O1^xxii^	48.22 (5)
O2—Cs1—O2^iv^	118.566 (14)	O1^xxi^—Cs2—O1^xxii^	48.22 (5)
O2^iii^—Cs1—O2^iv^	180.0	O2—Cs2—V1^vii^	86.53 (5)
O1^x^—Gd1—O1^vi^	180.00 (5)	O1^xvi^—Cs2—V1^vii^	147.92 (2)
O1^x^—Gd1—O1^xi^	88.78 (5)	O1^xvii^—Cs2—V1^vii^	147.92 (2)
O1^vi^—Gd1—O1^xi^	91.22 (5)	O1^xviii^—Cs2—V1^vii^	92.44 (2)
O1^x^—Gd1—O1^iv^	91.22 (5)	O1^iii^—Cs2—V1^vii^	92.44 (2)
O1^vi^—Gd1—O1^iv^	88.78 (5)	O1^xix^—Cs2—V1^vii^	29.680 (19)
O1^xi^—Gd1—O1^iv^	180.00 (6)	O1^i^—Cs2—V1^vii^	29.680 (19)
O1^x^—Gd1—O1^xii^	88.78 (5)	O1^xx^—Cs2—V1^vii^	121.61 (8)
O1^vi^—Gd1—O1^xii^	91.22 (5)	O1^xxi^—Cs2—V1^vii^	79.51 (4)
O1^xi^—Gd1—O1^xii^	88.78 (5)	O1^xxii^—Cs2—V1^vii^	79.51 (4)
O1^iv^—Gd1—O1^xii^	91.22 (5)	O2—Cs2—V1^iii^	86.53 (5)
O1^x^—Gd1—O1^ii^	91.22 (5)	O1^xvi^—Cs2—V1^iii^	92.44 (2)
O1^vi^—Gd1—O1^ii^	88.78 (5)	O1^xvii^—Cs2—V1^iii^	29.680 (19)
O1^xi^—Gd1—O1^ii^	91.22 (5)	O1^xviii^—Cs2—V1^iii^	147.91 (2)
O1^iv^—Gd1—O1^ii^	88.78 (5)	O1^iii^—Cs2—V1^iii^	29.681 (19)
O1^xii^—Gd1—O1^ii^	180.00 (6)	O1^xix^—Cs2—V1^iii^	147.92 (2)
O1^x^—Gd1—K1^xiii^	51.601 (16)	O1^i^—Cs2—V1^iii^	92.44 (2)
O1^vi^—Gd1—K1^xiii^	128.399 (17)	O1^xx^—Cs2—V1^iii^	79.51 (4)
O1^xi^—Gd1—K1^xiii^	51.602 (16)	O1^xxi^—Cs2—V1^iii^	121.61 (8)
O1^iv^—Gd1—K1^xiii^	128.398 (16)	O1^xxii^—Cs2—V1^iii^	79.51 (4)
O1^xii^—Gd1—K1^xiii^	117.93 (5)	V1^vii^—Cs2—V1^iii^	119.638 (11)
O1^ii^—Gd1—K1^xiii^	62.07 (5)	O2—V1—O1	109.06 (5)
O1^x^—Gd1—K1^vii^	128.399 (17)	O2—V1—O1^vi^	109.06 (5)
O1^vi^—Gd1—K1^vii^	51.601 (16)	O1—V1—O1^vi^	109.88 (5)
O1^xi^—Gd1—K1^vii^	128.398 (16)	O2—V1—O1^xxiii^	109.06 (5)
O1^iv^—Gd1—K1^vii^	51.602 (16)	O1—V1—O1^xxiii^	109.88 (5)
O1^xii^—Gd1—K1^vii^	62.07 (5)	O1^vi^—V1—O1^xxiii^	109.88 (5)
O1^ii^—Gd1—K1^vii^	117.93 (5)	O2—V1—K1^vii^	93.60 (4)
K1^xiii^—Gd1—K1^vii^	180.0	O1—V1—K1^vii^	157.34 (6)
O1^x^—Gd1—K1^xiv^	117.93 (5)	O1^vi^—V1—K1^vii^	60.518 (16)
O1^vi^—Gd1—K1^xiv^	62.07 (5)	O1^xxiii^—V1—K1^vii^	60.518 (16)
O1^xi^—Gd1—K1^xiv^	51.602 (16)	O2—V1—K1^iii^	93.60 (4)
O1^iv^—Gd1—K1^xiv^	128.398 (16)	O1—V1—K1^iii^	60.517 (16)
O1^xii^—Gd1—K1^xiv^	51.602 (16)	O1^vi^—V1—K1^iii^	60.518 (16)
O1^ii^—Gd1—K1^xiv^	128.398 (16)	O1^xxiii^—V1—K1^iii^	157.34 (6)
K1^xiii^—Gd1—K1^xiv^	102.28 (4)	K1^vii^—V1—K1^iii^	119.610 (10)
K1^vii^—Gd1—K1^xiv^	77.72 (4)	O2—V1—K1^xviii^	93.60 (4)
O1^x^—Gd1—K1^ix^	62.07 (5)	O1—V1—K1^xviii^	60.517 (16)
O1^vi^—Gd1—K1^ix^	117.93 (5)	O1^vi^—V1—K1^xviii^	157.34 (6)
O1^xi^—Gd1—K1^ix^	128.398 (16)	O1^xxiii^—V1—K1^xviii^	60.518 (16)
O1^iv^—Gd1—K1^ix^	51.602 (16)	K1^vii^—V1—K1^xviii^	119.610 (10)
O1^xii^—Gd1—K1^ix^	128.398 (16)	K1^iii^—V1—K1^xviii^	119.609 (10)
O1^ii^—Gd1—K1^ix^	51.602 (16)	O2—V1—K1^xiv^	180.0
K1^xiii^—Gd1—K1^ix^	77.72 (4)	O1—V1—K1^xiv^	70.94 (5)
K1^vii^—Gd1—K1^ix^	102.28 (4)	O1^vi^—V1—K1^xiv^	70.94 (5)
K1^xiv^—Gd1—K1^ix^	180.0	O1^xxiii^—V1—K1^xiv^	70.94 (5)
O1^x^—Gd1—K1^xv^	51.602 (16)	K1^vii^—V1—K1^xiv^	86.40 (4)
O1^vi^—Gd1—K1^xv^	128.398 (16)	K1^iii^—V1—K1^xiv^	86.40 (4)
O1^xi^—Gd1—K1^xv^	117.93 (5)	K1^xviii^—V1—K1^xiv^	86.40 (4)
O1^iv^—Gd1—K1^xv^	62.07 (5)	O2—V1—Cs1^xxv^	59.187 (4)
O1^xii^—Gd1—K1^xv^	51.602 (16)	O1—V1—Cs1^xxv^	49.87 (5)
O1^ii^—Gd1—K1^xv^	128.398 (16)	O1^vi^—V1—Cs1^xxv^	124.970 (19)
K1^xiii^—Gd1—K1^xv^	102.28 (4)	O1^xxiii^—V1—Cs1^xxv^	124.970 (19)
K1^vii^—Gd1—K1^xv^	77.72 (4)	K1^vii^—V1—Cs1^xxv^	152.79 (5)
K1^xiv^—Gd1—K1^xv^	102.28 (4)	K1^iii^—V1—Cs1^xxv^	66.64 (3)
K1^ix^—Gd1—K1^xv^	77.72 (4)	K1^xviii^—V1—Cs1^xxv^	66.64 (3)
O1^x^—Gd1—K1^iii^	128.398 (16)	K1^xiv^—V1—Cs1^xxv^	120.814 (4)
O1^vi^—Gd1—K1^iii^	51.602 (16)	O2—V1—Cs1^xxvi^	59.187 (4)
O1^xi^—Gd1—K1^iii^	62.07 (5)	O1—V1—Cs1^xxvi^	124.969 (19)
O1^iv^—Gd1—K1^iii^	117.93 (5)	O1^vi^—V1—Cs1^xxvi^	124.970 (19)
O1^xii^—Gd1—K1^iii^	128.398 (16)	O1^xxiii^—V1—Cs1^xxvi^	49.87 (5)
O1^ii^—Gd1—K1^iii^	51.602 (16)	K1^vii^—V1—Cs1^xxvi^	66.65 (3)
K1^xiii^—Gd1—K1^iii^	77.72 (4)	K1^iii^—V1—Cs1^xxvi^	152.78 (5)
K1^vii^—Gd1—K1^iii^	102.28 (4)	K1^xviii^—V1—Cs1^xxvi^	66.65 (3)
K1^xiv^—Gd1—K1^iii^	77.72 (4)	K1^xiv^—V1—Cs1^xxvi^	120.813 (4)
K1^ix^—Gd1—K1^iii^	102.28 (4)	Cs1^xxv^—V1—Cs1^xxvi^	96.109 (5)
K1^xv^—Gd1—K1^iii^	180.0	O2—V1—Cs1	59.187 (4)
O2—K1—O1^xvi^	96.52 (6)	O1—V1—Cs1	124.969 (19)
O2—K1—O1^xvii^	96.52 (6)	O1^vi^—V1—Cs1	49.87 (5)
O1^xvi^—K1—O1^xvii^	63.65 (4)	O1^xxiii^—V1—Cs1	124.970 (19)
O2—K1—O1^xviii^	96.52 (6)	K1^vii^—V1—Cs1	66.65 (3)
O1^xvi^—K1—O1^xviii^	55.49 (4)	K1^iii^—V1—Cs1	66.65 (3)
O1^xvii^—K1—O1^xviii^	118.73 (2)	K1^xviii^—V1—Cs1	152.78 (5)
O2—K1—O1^iii^	96.52 (6)	K1^xiv^—V1—Cs1	120.813 (4)
O1^xvi^—K1—O1^iii^	118.73 (2)	Cs1^xxv^—V1—Cs1	96.109 (5)
O1^xvii^—K1—O1^iii^	55.49 (4)	Cs1^xxvi^—V1—Cs1	96.109 (5)
O1^xviii^—K1—O1^iii^	166.32 (11)	O2—V1—Cs2	0.0
O2—K1—O1^xix^	96.52 (6)	O1—V1—Cs2	109.06 (5)
O1^xvi^—K1—O1^xix^	118.73 (2)	O1^vi^—V1—Cs2	109.06 (5)
O1^xvii^—K1—O1^xix^	166.32 (11)	O1^xxiii^—V1—Cs2	109.06 (5)
O1^xviii^—K1—O1^xix^	63.65 (4)	K1^vii^—V1—Cs2	93.60 (4)
O1^iii^—K1—O1^xix^	118.73 (2)	K1^iii^—V1—Cs2	93.60 (4)
O2—K1—O1^i^	96.52 (6)	K1^xviii^—V1—Cs2	93.60 (4)
O1^xvi^—K1—O1^i^	166.32 (11)	K1^xiv^—V1—Cs2	180.0
O1^xvii^—K1—O1^i^	118.73 (2)	Cs1^xxv^—V1—Cs2	59.187 (4)
O1^xviii^—K1—O1^i^	118.73 (2)	Cs1^xxvi^—V1—Cs2	59.187 (4)
O1^iii^—K1—O1^i^	63.65 (4)	Cs1—V1—Cs2	59.187 (4)
O1^xix^—K1—O1^i^	55.49 (4)	V1—O1—Gd1^xxv^	162.94 (8)
O2—K1—V1^vii^	86.40 (4)	V1—O1—K1^xviii^	89.81 (3)
O1^xvi^—K1—V1^vii^	147.94 (2)	Gd1^xxv^—O1—K1^xviii^	92.19 (4)
O1^xvii^—K1—V1^vii^	147.94 (2)	V1—O1—K1^iii^	89.81 (3)
O1^xviii^—K1—V1^vii^	92.45 (2)	Gd1^xxv^—O1—K1^iii^	92.19 (4)
O1^iii^—K1—V1^vii^	92.45 (2)	K1^xviii^—O1—K1^iii^	166.32 (11)
O1^xix^—K1—V1^vii^	29.676 (19)	V1—O1—Cs1^xxv^	105.95 (5)
O1^i^—K1—V1^vii^	29.676 (19)	Gd1^xxv^—O1—Cs1^xxv^	91.12 (4)
O2—K1—V1^iii^	86.40 (4)	K1^xviii^—O1—Cs1^xxv^	83.48 (5)
O1^xvi^—K1—V1^iii^	92.45 (2)	K1^iii^—O1—Cs1^xxv^	83.48 (5)
O1^xvii^—K1—V1^iii^	29.675 (19)	V1—O2—Cs2	180.0
O1^xviii^—K1—V1^iii^	147.94 (2)	V1—O2—K1	180.0
O1^iii^—K1—V1^iii^	29.676 (19)	Cs2—O2—K1	0.0
O1^xix^—K1—V1^iii^	147.94 (2)	V1—O2—Cs1^xxv^	96.93 (3)
O1^i^—K1—V1^iii^	92.45 (2)	Cs2—O2—Cs1^xxv^	83.07 (3)
V1^vii^—K1—V1^iii^	119.610 (10)	K1—O2—Cs1^xxv^	83.07 (3)
O2—K1—V1^xviii^	86.40 (4)	V1—O2—Cs1^xxvi^	96.93 (3)
O1^xvi^—K1—V1^xviii^	29.675 (19)	Cs2—O2—Cs1^xxvi^	83.07 (3)
O1^xvii^—K1—V1^xviii^	92.45 (2)	K1—O2—Cs1^xxvi^	83.07 (3)
O1^xviii^—K1—V1^xviii^	29.676 (19)	Cs1^xxv^—O2—Cs1^xxvi^	118.566 (13)
O1^iii^—K1—V1^xviii^	147.94 (2)	V1—O2—Cs1	96.93 (3)
O1^xix^—K1—V1^xviii^	92.45 (2)	Cs2—O2—Cs1	83.07 (3)
O1^i^—K1—V1^xviii^	147.94 (2)	K1—O2—Cs1	83.07 (3)
V1^vii^—K1—V1^xviii^	119.610 (10)	Cs1^xxv^—O2—Cs1	118.566 (13)
V1^iii^—K1—V1^xviii^	119.609 (10)	Cs1^xxvi^—O2—Cs1	118.566 (13)
O2—K1—V1^xx^	180.0		

Symmetry codes: (i) *x*−*y*+1, *x*, −*z*+1; (ii) −*x*+*y*−1, −*x*, *z*; (iii) −*x*, −*y*+1, −*z*+1; (iv) *x*, *y*−1, *z*; (v) *y*−1, −*x*+*y*−1, −*z*+1; (vi) −*y*+1, *x*−*y*+1, *z*; (vii) −*x*+1, −*y*+1, −*z*+1; (viii) *x*−1, *y*−1, *z*; (ix) −*x*, −*y*, −*z*+1; (x) *y*−1, −*x*+*y*−1, −*z*; (xi) −*x*, −*y*+1, −*z*; (xii) *x*−*y*+1, *x*, −*z*; (xiii) *x*−1, *y*−1, *z*−1; (xiv) *x*, *y*, *z*−1; (xv) *x*, *y*−1, *z*−1; (xvi) *x*−*y*+1, *x*+1, −*z*+1; (xvii) *y*−1, −*x*+*y*, −*z*+1; (xviii) −*x*+1, −*y*+2, −*z*+1; (xix) *y*, −*x*+*y*, −*z*+1; (xx) *x*, *y*, *z*+1; (xxi) −*x*+*y*, −*x*+1, *z*+1; (xxii) −*y*+1, *x*−*y*+1, *z*+1; (xxiii) −*x*+*y*, −*x*+1, *z*; (xxiv) *x*, *y*+1, *z*+1; (xxv) *x*, *y*+1, *z*; (xxvi) *x*+1, *y*+1, *z*.
